# The Association between Body Composition using Dual energy X-ray Absorptiometry and Type-2 Diabetes: A Systematic Review and Meta-Analysis of Observational studies

**DOI:** 10.1038/s41598-019-49162-5

**Published:** 2019-09-02

**Authors:** Preeti Gupta, Carla Lanca, Alfred T. L. Gan, Pauline Soh, Sahil Thakur, Yijin Tao, Neelam Kumari, Ryan E. K. Man, Eva K. Fenwick, Ecosse L. Lamoureux

**Affiliations:** 1 Singapore Eye Research Institute, Singapore and Singapore National Eye Centre, Singapore, Singapore; 2 Duke-NUS Medical School, Singapore, Singapore

**Keywords:** Epidemiology, Type 2 diabetes

## Abstract

The association between objective measures of body composition (BC) with type 2 diabetes (T2DM) is inconclusive. We conducted a systematic review and meta-analysis to examine the association between several body composition (BC) indices assessed using dual energy X-ray absorptiometry (DXA), and T2DM. Using PRISMA guidelines, we searched for observational studies investigating BC measures, including total body fat mass (BFM), visceral fat mass (VFM), subcutaneous fat mass (SFM), and fat free mass (FFM); and T2DM. Of 670 titles initially identified, 20 were included. High VFM was consistently associated with T2DM. For every kg increase in VFM, the odds of having T2DM increased by two-fold for males (OR 2.28 [95% CI 1.42 to 3.65], p = 0.001) and more than 4-fold for females (OR 4.24 [1.64 to 11.02], p = 0.003). The presence of T2DM was associated with 2-fold higher odds of low FFM (OR 2.38 [1.44 to 3.95]). We found evidence that greater VFM is a risk factor for prevalent and incident T2DM. While the presence of T2DM is associated with reduced FFM; the relationship between FFM and BFM with T2DM remains unclear. Reducing VFM and increasing FFM through lifestyle changes may reduce the risk of T2DM and mitigate its deleterious effect on BC, respectively.

## Introduction

The prevalence of diabetes mellitus (DM) is rapidly increasing worldwide, with 592 million people expected to have the condition by 2035^1^. As such, the prevention and management of DM has become a crucial public health concern with an emphasis on addressing modifiable risk factors, such as poor diet, sedentary lifestyle, and obesity^2^.

While the relationship between obesity and DM, using surrogate anthropometric measures such as body mass index [BMI]^3,4^, waist circumference [WC]^5,6^, and waist-hip ratio [WHR]^7,8^, have been widely studied, results have been equivocal^9,10^, as these proxy measures are unable to distinguish body composition (BC) indices such as fat tissue deposition from muscle mass and bone density. Furthermore, these surrogate measures do not provide information on the location of fat mass deposition, emphasizing the need to evaluate the contribution of objective components of BC on DM risk.

Specific BC measurements can be estimated indirectly using various objective techniques such as air displacement plethysmography (BodPod), bioelectrical impedance analysis (BIA)^11^, and dual energy X-ray absorptiometry (DXA). However, BC profiles measured by different objective techniques are not interchangeable, as studies have reported poor concordance between these different methods^12–14^. In this analysis we only included studies that assessed BC measures using DXA, which is commonly used^15^, and is an acceptable technique for measuring BC in clinical studies^16,17^ with a substantially lower cost, no complex post processing requirements, and minimal radiation exposure (~1 µSV) compared to computed tomography (CT) or magnetic resonance imaging (MRI) scan^18^. There is increasing evidence that DXA-assessed BC measures are associated with the onset, progression, treatment response, and health outcomes of cardiometabolic diseases, including type 2 DM (T2DM)^19–22^.

To date, there is no comprehensive review on the relationship between DXA-assessed BC measures, including total body fat mass (BFM), visceral fat mass (VFM), subcutaneous fat mass (SFM), muscle mass (MM), and the presence and incidence of DM. A better understanding of this association is critical as it can inform clinical guidelines and interventions for the management of DM. We therefore performed a systematic review and meta-analysis of observational studies evaluating the bidirectional associations between objectively assessed BC measures (i.e. BFM including VFM and SFM; and MM) and the prevalence or incidence of T2DM. We also identified key knowledge gaps and suggest future research directions.

## Methods

### Literature search

We performed a systemic review and comprehensive literature search using three sources (PubMed, Web of Science and the Cochrane Central Register of Controlled Trials) for English language research articles published between January 1998 and August 2018. The databases were systematically searched using a combination of the following keywords and Boolean operators: body composition OR body fat mass OR visceral fat mass OR subcutaneous fat mass OR adiposity OR visceral adipose tissue OR fat free mass OR muscle mass OR lean body mass OR skeletal muscle mass AND type 2 diabetes. The studies included observational epidemiological studies in T2DM populations such as cohort, case control and cross-sectional studies, in which DXA was utilized to assess BC in humans. This process continued until a search saturation point was found; i.e. the point at which additional terms showed no improvement in our search result. Relevant references identified from the bibliographies of pertinent articles were also retrieved.

### Study selection

Using our search strategy, 670 titles were initially identified. Two authors (PG and CL) assessed the titles independently according to predefined inclusion criteria. Studies were then systematically excluded after detailed examination, if the title and abstract were not relevant. Any potential disagreements were resolved through consulting the senior author (EL). If necessary, full-text articles of studies were also obtained, particularly if there was insufficient information within the abstract to determine exclusion.

#### Inclusion criteria

Eligibility criteria were based on the PICOS (participants, intervention, comparability, outcomes, study design) framework recommended by the PRISMA guidelines^23^.

1. **Study Type:** We included observational studies (cross-sectional, case-control and prospective).
2. **Participants:** Studies that included human participants with T2DM.
3. **Exposures:** Four objective BC measurements (total BFM, VFM, SFM, and MM) obtained using DXA were selected as the exposures of interest. For MM, we included studies which reported total lean mass (TLM) or appendicular skeletal muscle mass (ASM) – two of the most commonly used measures of MM. Furthermore, we accepted studies reporting on BFM or MM and DM as *either* exposure/outcome.
4. **Outcomes:** Outcomes were the prevalence or incidence of T2DM. We accepted studies using different T2DM assessment methods, including but not limited to: random glucose ≥11.1 mmol/L, HbA1c ≥6.5% (≥48 mmol/moL), fasting plasma glucose >125 mg/dl or ≥7.0 mmol/l, self-reported use of oral hypoglycaemic medications or insulin, and/or history of physician-diagnosed diabetes.

#### Exclusion criteria

The following types of papers were excluded:

1. Conference abstracts.
2. Papers not written in the English language.
3. Studies on animals, and *in-vitro*/*in-vivo* studies.
4. Studies involving individuals with type 1 DM.
5. Studies in people without diabetes, or those with participants with impaired glucose tolerance or pre-diabetes.
6. Studies measuring biomarkers of BC in serum, blood, or urine without any link to BC assessment using DXA.
7. Exposures which were assessed using objective methods other than DXA, such as bioelectrical impedance analysis (BIA), MRI or CT.
8. Studies only measuring outcomes of “cardiometabolic diseases” or “metabolic risk factors” without specific reference to T2DM.
9. Articles with insufficient data to draw conclusions. This included any form of data insufficiency which did not enable us to draw conclusions from/evaluate the study, (e.g. lack of exposure/outcome definitions, or lack of statistical analysis).
10. For our meta-analysis, we also excluded studies that did not report the required statistical parameters and which we could not obtain despite repeated attempts to contact the study authors.

### Data extraction

A standardized data extraction form based on the “Strengthening the Reporting of Observational Studies in Epidemiology” (STROBE) statement^24^ was used to extract the following relevant data from each included article: authors, year, study design, sample size, population characteristics, age of participants, objective BC measures, DM outcome type, method of T2DM diagnosis, adjustment for confounders used in analysis, statistical methods used, and summary of key findings. Data extraction was done by one author (PG) and confirmed by another (CL).

### Study quality evaluation

The quality of observational studies was assessed using a modified version of the Newcastle Ottawa Scale (NOS), a validated tool for evaluating observational study designs^25^. Originally designed to assess prospective and case-control studies, an adapted version of the NOS was used in the current study to allow the assessment of cross-sectional studies^26^. The NOS uses three main bias-reducing criteria to award up to a maximum of 9 stars: (a) the selection and representativeness of the participants (maximum of 4 stars), (b) the comparability of groups (maximum of 2 stars), and (c) the ascertainment of exposure (for case-control) or outcome (for prospective and cross-sectional) (maximum of 3 stars). Given that BMI or WC are important confounders in the association between BC and T2DM, we gave studies a star (under comparability criteria) if they adjusted for BMI or WC in their analyses. Studies assigned 0–4, 5–6, and ≥7 stars were considered as low, medium and high quality (low risk of bias) respectively. Studies with low quality (i.e. high risk of bias) were excluded from this review.

### Statistical analysis

All statistical analyses were performed using Intercooled Stata version 15 for Windows (StataCorp, Lake Station, TX). Due to the paucity of studies reporting on the total BFM-T2DM relationship, and no studies on SFM, we could not perform meta-analysis of these BC measures and T2DM. We conducted random effects meta-analysis to pool the crude odds ratios (ORs) relating VFM and MM with T2DM, including studies that provided this information. ORs were calculated from two-way contingency tables where they were not reported but cell counts were available. We chose not to pool adjusted ORs as studies varied substantially in their adjustment for potential confounders which might have affected the direction and significance of the associations reported. Given that BC varies by gender, the meta-analysis of VFM-T2DM was stratified by gender. In contrast, no stratification was done for the meta-analysis of MM-T2DM as too few studies provided gender-stratified data. Heterogeneity was quantified using the I-squared statistic. A higher I-squared value meant greater heterogeneity in study effects. We reported all pooled estimates with 95% confidence intervals and judged a p-value < 0.05 as statistically significant.

## Results

### Description of studies

**Figure 1:**
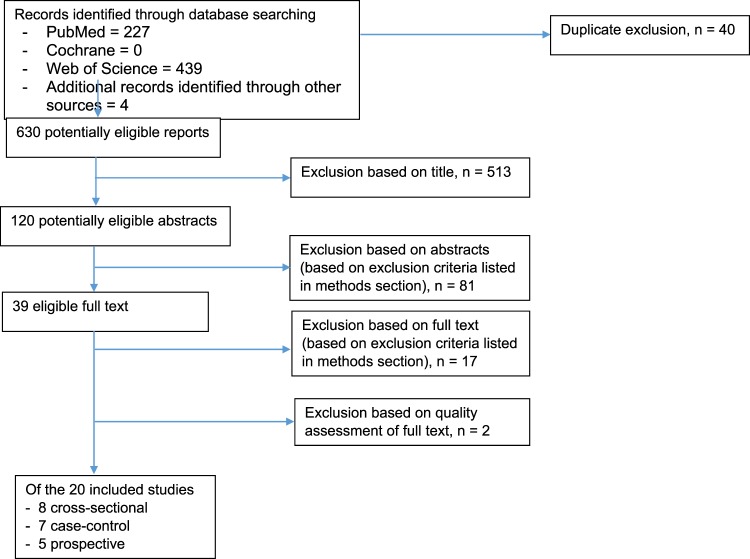
PRISMA Flow Diagram: Selection of included studies.

Of 670 titles screened, 120 were extracted for detailed evaluation, of which 20 adhered to our inclusion criteria (Fig. 1). They comprised five prospective, eight cross-sectional, and seven case-control studies. The majority had high NOS scores, with 19 classified as “high quality” (≥7 stars) and one as “moderate quality” (5 stars). No study was excluded because of “low quality” (Tables 1 and 2).

**Table 1:** Summary of data extracted from the eight observational studies on the association between total, and visceral fat mass and diabetes included in the systematic review.

Author, year	Association	Study design	Quality	Study population	Sample size	DM (outcome) assessment method	Analysis and variables adjusted for	Main findings
Choi, 2017	Total body fat % was higher in individuals with T2DM	Cross-sectional	8	Asians (Korean)	6575 (3027 males, 3548 postmenopausal females)	Fasting glucose ≥126 mg/dL (7.0 mmol/L), medical diagnosis, use of oral hypoglycaemic medications or insulin injections	T-test	Total body fat % was higher in both men (23.44 ± 4.91 vs 21.98 ± 5.23, p < 0.001) and women (34.85 ± 5.05 vs 33.85 ± 5.59, p < 0.001) with T2DM than those without.
Neeland, 2012	No difference in total fat mass (kg) and body fat %	Prospective	8	Caucasians (United States)	732 (256 men, 476 women)	Prevalent medical treatment for T2DM, fasting blood glucose ≥126 mg/dL (7.0 mmol/L),or non-fasting blood glucose ≥200 mg/dl	Chi square and Wilcoxon rank-sum test	No difference in total fat mass (35.3 vs 35.5, p = 0.51) and body fat % (39.8 vs 40.4, p = 0.51) among those with incident T2DM compared to no DM
Raska, 2016	No difference in fat mass/height^2^ and fat mass %	Case-control	5	Caucasians (Australians)	139 postmenopausal women (68 with T2DM, 71 age-and weight-matched without T2DM)	Biochemical analyses of HbA1c and serum fasting glucose	T-test Age-and weight-matched	No difference in fat mass/height^2^ (14.4 ± 5.5 vs 13.35 ± 5.2), and fat mass % (41.12 ± 4.9 vs 41.19 ± 5.97) in postmenopausal women with and without T2DM
Stoney, 1998	No difference in total fat mass	Case-control	8	Caucasians	84 postmenopausal women (42 T2DM, 42 non-DM, age and BMI matched)	Current DM medication or abnormal OGTT	T-test Age and BMI	No difference in total fat mass (30.9 ± 1.5 vs 31.8 ± 1.5) in postmenopausal women with and without T2DM
Heshka, 2008	T2DM was associated with reduced total BFM	Cross-sectional	7	Caucasians (Black, White and Hispanics)	1315 (490 men, 825 women)		Multivariable Regression Age, gender, race, clinical site, height, weight and body size	Less total fat mass (−1.4 ± 0.3 [S.E.]; 34.5 vs 35.8 kg, p < 0.001) in T2DM than controls
Nordstrom, 2016	Higher VFM increased the risk of T2DM	Cross-sectional	7	Caucasians (Swedish)	1393 (705 men, 688 women)	Fasting-plasma glucose level of at least 7 mmol/L or based on questionnaire responses	Logistic Regression Smoking, PA, SBP, DBP, cholesterol, HDL, TG, previous MI and stroke	Per SD increase in VFM/body weight was the strongest predictor of T2DM in men (OR = 3.64, 95% CI: 2.53–5.25).
Rothney, 2013	Higher VFM increased the risk of T2DM	Cross-sectional	7	Caucasians (Italian)	939 (541 men, 398 women)	Fasting plasma glucose >125 mg/dl	Multivariable Linear Regression Age, BMI and WC	The OR (per SD change in VFM) for T2DM = 2.07 (95%CI: 0.73–5.87) for women and 2.25 (95% CI: 1.21–4.19) for men.
Jung, 2016	Higher VFM increased the risk of T2DM	Cross-sectional	7	Asians (Korean)	1603 (611 men, 992 women)	Fasting glucose ≥126 mg/dL (7.0 mmol/L), 2-h glucose ≥200 mg/dL (11.1 mmol/L) during the OGTT (75 g), HbA1c level ≥6.5%, or use of hypoglycaemic medications	Multivariable Logistic Regression Age, alcohol consumption, PA, education and menopause (for females)	VFM in the upper 10^th^ percentile had highest OR for DM (men: OR = 15.9, 95%CI: 6.4–39.2; women: OR = 6.9, 95%CI: 3.5–13.7). VFM had the highest AUC with DM (men: 0.69, 95% CI: 0.64–0.73; women: 0.70, 95%CI: 0.67–0.74).

T2DM: type-2 diabetes, DM: diabetes mellitus; BFM: body fat mass; VFM: visceral fat mass; FFM: fat free mass; ASM: appendicular skeletal mass; BMI: body mass index; WC: waist circumference; PA: physical activity; SBP: systolic blood pressure; DBP: diastolic blood pressure; TG: triglyceride; HDL: high density cholesterol; LDL: low density cholesterol; HRT: hormone replacement therapy; MI: myocardial infarction; OR: odds ratio; CI: confidence interval; HR: hazard ratio; SD: standard deviation; AUC: area under curve.

**Table 2:** Summary of data extracted from the 12 observational studies on the association between fat free mass and diabetes included in the systematic review.

Author, year	Association	Study Design	Quality	Study Population	Sample Size	DM outcome assessment	Analysis and variables adjusted for	Main Findings
Kim, 2014	Low muscle mass measures in men with T2DM	Case-control	7	Asians (Koreans)	414 (189 men, 225 women); 144 T2DM, 270 controls	Onset of DM after age 25 years, use of oral hypoglycaemic medications or insulin, or fasting plasma glucose ≥126 mg/dL.	Multivariable Logistic Regression Age, BMI, current smoking, SBP, DBP, total cholesterol, TG, HDL	Older men with T2DM had 2–4 times increased risk of low muscle mass measures (ASM/height^2^, ASM/weight and total skeletal muscle/weight; OR range = 2.63–4.45).
Kim, 2010 (KSOS)	Low ASM/height^2^ in T2DM	Case-control,	8	Asians (Koreans)	810 (414 DM, 396 controls)	Not defined	Multivariable Logistic Regression Age, gender, BMI, smoking, alcohol, PA, SBP, DBP, medications and lipid profile	T2DM had higher risk of low ASM/height^2^ (OR = 3.06, 95% CI: 1.42–6.62) than those without.
Anbalagan, 2013	Low ASM/height^2^ in T2DM	Case-control	8	Asians (Indians)	152 (72 T2DM and 72 age-sex matched controls)	Fasting plasma glucose and 2-h post load (75 g) plasma glucose	Multivariable Logistic Regression Age, gender, diet, PA, smoking, alcohol, DM	T2DM was associated with increased risk of low ASM/height^2^ (OR = 6.01, 95% CI: 1.34–26.88), compared to those without. The relationship became insignificant on further adjustment for HbA1c or fasting plasma glucose (OR = 3.29, 95% CI: 0.629–17.28 and OR = 3.94, 95% CI: 0.794–19.65, respectively).
Guerroro, 2016	Lower ASM/BMI in women with T2DM	Case-control	7	Caucasians (United states)	139 adults (88 women, 51 men); 100 T2DM and 39 controls	Selected T2DM patients with over 4 years on oral anti diabetic drugs or insulin	T-test or Mann Whitney	Women with T2DM had significantly lower ASM/BMI (5.3 [4.4–8]) than those without (5.9 [4.2–8]; p = 0.02).
Moon, 2014	Low ASM/weight in non-obese T2DM adults	Cross-sectional	7	Asians (Koreans)	10432 adults (4558 men, 5874 women)	Previously diagnosed T2DM, use of anti-hyperglycaemic medication, or fasting plasma ≥100 mg/dL	Multivariable Logistic Regression Age, sex, region, smoking, alcohol consumption, exercise, family income and BMI	In older (≥60 yrs) non-obese, those with low ASM/weight had higher risk of T2DM (OR = 2.44, 95% CI: 1.69–3.53, p < 0.001). This was not significant in obese (OR = 1.26, 95% CI: 0.76–2.10, p = 0.362) individuals.
Yoon, 2016	No association between ASM/height^2^ and T2DM	Cross-sectional	7	Asians (Koreans)	269 men (79 T2DM, 190 controls)	HbA1c ≥6.5% or current use of insulin or oral hypoglycaemic medication	T-test Age, smoking, alcohol, PA, BMI, duration of DM	No significant difference in ASM/height^2^ between subjects with or without T2DM (7.46 ± 0.77 vs 7.39 ± 0.85, p = 0.563).
Akeroyd, 2014	Lower ASM in T2DM	Cross-sectional	8	Caucasians (United states)	1137 men (142 T2DM, 995 controls)	Self-report of physician diagnosed or use of oral hypoglycaemic agents	Multivariable Linear Regression Age, race, BMI, PA	Men with T2DM had significantly lower ASM (mean deviation [MD] = −1.04 kg, p = 0.04) than those without. No significant difference in leg lean mass.
Davidson, 2014	Lower FFM in T2DM	Cross-sectional	7	Caucasians (United states)	171 (95 T2DM, 76 controls)	Physician diagnosis	General linear models Height, weight, age, sex and race	Adjusted FFM was significantly lower in those with T2DM than controls (p < 0.05)
Larsen, 2016: The Health ABC study	Greater FFM is associated with lower incidence of DM for older normal-weight women but not for men or overweight women.	Prospective	8	Caucasians (United states)	2076 (202 incident T2DM); 958 men, 20176 women	Physician diagnosed, use of oral hypoglycaemic agents or insulin with onset after age 25 years, fasting plasma glucose ≥7.0 mmol/L	Cox Regression Age, race, clinical site, PA, smoking, lipid profile, hypertension and VFM	High FFM was not associated with lower risk of incident T2DM (HR = 0.37, 95%CI: 0.17–0.83) in normal weight women. Higher levels of the FFM was associated with greater risk of incident T2DM for overweight/obese (total FFM: HR = 1.10, 95%CI: 0.89–1.36) women. No associations seen in men
Park, 2009, The Health ABC study	T2DM is associated with excessive loss of total muscle mass and ASM	Prospective	7	Caucasians (United states)	2675 (1324 men, 1351 women); 628 T2DM, 20147 controls	Physician diagnosed, use of oral hypoglycaemic agents or insulin with onset after age 25 years, fasting plasma glucose ≥7.0 mmol/L, or a 2-h post challenge plasma glucose ≥11.1 mmol/l	Generalized Estimating Equation Age, sex, race, clinic site, baseline BMI, weight loss	The rate of decline in total muscle mass (−186 ± 25 vs −125 ± 7, p < 0.05) and ASM (−149 ± 14 vs −113 ± 4, p < 0.05) was greater in older adults with undiagnosed T2DM, than in those without.
Li, 2016	Reduced FFM & ASM/ht^2^ is not a risk factor for incident T2DM in men	Prospective	8	Caucasians (Australians)	1632 men (146 incident T2DM, 1486 controls)	Previous doctor diagnosis, antiglycemic medication use, fasting plasma glucose ≥7.0 mmol/L (≥126 mg/dl), or HbA1c ≥6.5% (48 mmol/mol).	Multivariable Logistic Regression Age, income, cohort, WC, fasting plasma glucose, PA, hypertension, TG, family history of DM, and grip strength	Reduced FFM & ASM/ht^2^ were not significant risk factors for T2DM incidence (per 5 kg unit increase in FFM and per 1 kg/m^2^ increase in ASM/ht^2^ on T2DM incidence, OR = 1.03, 95% CI:0.87–1.24; OR = 1.08: 95% CI: 0.83–1.39, respectively).
Renoud, 2014	T2DM is associated with faster muscle loss in older men	Prospective	7	Caucasians (French)	608 men	Glycemia ≥100 mg/dL	Multivariable Linear Regression Age, testosterone and PA	Men with T2DM had higher age-related acceleration of muscle loss versus men without (−0.08 vs -0.03%/year/age, p < 0.05)

T2DM: type-2 diabetes, DM: diabetes mellitus; VFM: visceral fat mass; FFM: fat free mass; ASM: appendicular skeletal mass; BMI: body mass index; WC: waist circumference; PA: physical activity; SBP: systolic blood pressure; DBP: diastolic blood pressure; TG: triglyceride; HDL: high density cholesterol; OR: odds ratio; CI: confidence interval.

### Association of BFM and its components with T2DM

#### Total BFM as the exposure

The association between total BFM and T2DM was equivocal^27–30^. In a cross-sectional study of Korean adults aged 50 years or older, Choi and colleagues reported increased total body fat % in both men (23.44 ± 4.91 vs 21.98 ± 5.23, p < 0.001) and postmenopausal women (34.85 ± 5.05 vs 33.85 ± 5.59, p < 0.001) with T2DM compared to those without^29^. In contrast, in a longitudinal study, Neeland and associates found no association between total BFM or body fat % and incident T2DM^30^. However, as both studies used simple univariate methods, the lack of multivariate adjustment for potential confounders, may bias their results.

#### Total BFM as the outcome

We found only one cross-sectional study of 1315 Caucasians (mean age 58.5 ± 6.6), published study by Heska and colleagues, who reported that subjects with T2DM had reduced total BFM, compared to those without (34.5 vs 35.8 kg, p < 0.001)^20^.

#### VFM as the exposure

We found evidence of a strong association between accumulation of VFM and prevalent T2DM, after adjusting for relevant confounders^31–35^. In a cross-sectional sample of 939 Italian men and women, VFM was independently associated with a two-fold risk of having T2DM, even after adjustment for BMI and WC^35^. Similarly, Nordstrom and colleagues demonstrated almost a four-fold (OR = 3.64, 95% CI: 2.53–5.25) and 1.5-fold (OR = 1.41, 95% CI: 0.93–2.13) higher odds of having T2DM in men and women for every SD increase in VFM/body weight, respectively^33^.

#### Subcutaneous Fat Mass (SFM) as the exposure or outcome

We found no published data on the relationship between SFM (assessed using DXA) and T2DM.

### Association of FFM with T2DM

#### MM as the outcome

Of twelve relevant studies, MM was the outcome in nine of them^21,36–46^. Of these, eight (five case control, one cross-sectional, and two longitudinal) found an independent association between T2DM and reduced MM^36–43^. For instance, in a case-control study, Kim and associates, reported that even after adjusting for relevant covariates older (>65 years) Korean men with T2DM had a 2–4 fold increased odds of low muscle mass measures (ASM/height^2^, ASM/weight, and TSM/weight)^40^. Similarly, Anbalagan and colleagues found that Asian Indians with T2DM had significantly increased odds of low ASM/ht^2^ (OR = 6.01, 95% CI: 1.34–26.88) than those without^37^, after adjusting for age, gender, diet, physical activity, smoking, alcohol, DM duration and treatment, although the relationship was attenuated when HbA1c was introduced into the model (OR = 3.94, 95%CI: 0.79–19.65).

In the Health, ABC Study of community-dwelling older adults (70–79 years), Parks and colleagues found that the rate of decline in TLM and ASM (gram/year) was most profound in older adults with undiagnosed T2DM (TLM: −186 ± 25; ASM: −149 ± 14), followed by diagnosed T2DM (TLM: −106 ± 20; ASM: −130 ± 11) than in those without DM (TLM: −125 ± 7; ASM: −113 ± 4), even after adjusting for changes in body weight over time^42^.

#### MM as the exposure

The association between MM and T2DM was equivocal^21,45,46^. For instance, in the Korean National Health and Nutrition Examination Survey (KNHANES), low muscle mass was found to be an early marker for T2DM (individuals with low ASM/wt had higher odds of T2DM (OR = 2.44, 95% CI: 1.69–3.53; p < 0.001) than those without)^21^. Conversely, in a fully-adjusted prospective, population-based, randomly selected cohort of men (mean age 54.1 ± 11.4 years), Li and associates found that reduced TLM and ASM/ht^2^ were not significant risks factors for incident T2DM (per 5 kg increase in TLM and per 1 kg/m^2^ increase in ASM/ht^2^ on T2DM incidence, OR = 1.03, 95% CI: 0.87–1.24; OR = 1.08: 95% CI: 0.83–1.39, respectively)^46^.

### Meta-analysis of the association of VFM with T2DM

#### VFM as the exposure

**Figure 2:**
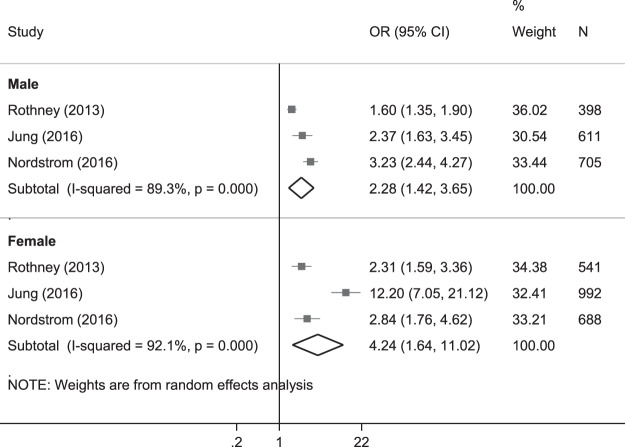
Forest plot* of the crude odds ratio of diabetes per 1 kg increase in visceral fat mass. *The size of the box of each study effect corresponds to the relative weight given to that study in the meta-analysis; the diamond refers to the overall pooled estimates with 95% confidence interval.

For the VFM-T2DM relationship, three cross-sectional studies were included in the meta-analysis^31,33–35^. We found that for every kg increase in VFM, the odds of T2DM increased more than two-fold for males (OR = 2.28, 95% CI: 1.42 to 3.65, p = 0.001) and more than 4-fold for females (OR = 4.24, 95% CI:1.64 to 11.02, p = 0.003; Fig. 2).

### Meta-analysis of the association of MM with T2DM

**Figure 3:**
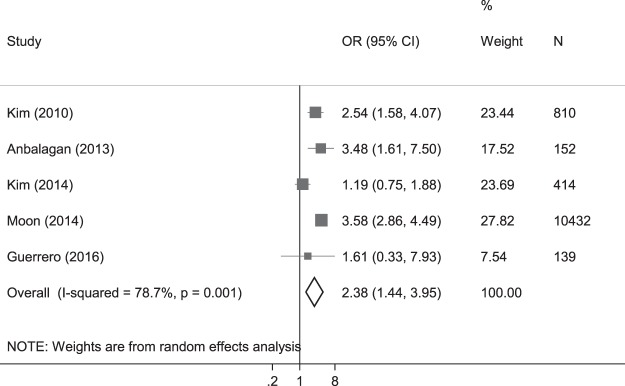
Forest plot* of the crude odds ratio of diabetes presence to low appendicular skeletal muscle mass. *The size of the box of each study effect corresponds to the relative weight given to that study in the meta-analysis; the diamond refers to the overall pooled estimates with 95% confidence interval.

Estimates in the form of ORs were available from four studies with low MM as the outcome^37,39–41^ and one study with T2DM as the outcome^21^. In line with our systematic review, our meta-analysis presented ORs of MM. We found presence of T2DM was associated with 2.4 times higher odds of low MM (ASM/ht^2^; crude OR 2.38 [1.44 to 3.95], p = 0.001; Fig. 3).

## Discussion

In this systematic review and meta-analysis of the relationship between DXA- measured BC indices and T2DM, we found a consistent evidence of an association between high levels of visceral fat and the risk of T2DM, as well as a significant association between T2DM presence and low MM, after accounting for traditional diabetes risk factors. These findings suggest that reducing VFM and improving MM through lifestyle changes such as diet and physical activity, may reduce the risk of T2DM, and mitigate the deleterious effect of the condition on MM. In contrast, the relationship between total BFM, MM and T2DM, remains unclear, while no studies to date have explored the relationship between DXA-assessed SFM and T2DM. Longitudinal studies are however needed to better understand the temporality and mechanisms underlying the relationships between total BFM, SFM and MM; and T2DM.

Our finding that VFM may increase the risk of T2DM^31,33,35^ could be explained by several potential underlying mechanisms. First, increased VFM is related to low adiponectin levels^47^. Adiponectin plays a pivotal role in energy metabolism, and has antiglycemic (increases insulin sensitivity), anti-inflammatory, antiangiogenic, and cardio protective properties^48,49^. Therefore, reduced secretion of adiponectin in individuals with high fat mass, particularly VFM, may lead to a cascade of biochemical reactions including an increase in insulin resistance and impaired glucose homeostasis (by augmenting hepatic gluconeogenesis and inhibiting glucose uptake in skeletal muscles)^50^ resulting in hyperglycaemia. Second, lower adiponectin levels increases the secretion of pro-inflammatory cytokines (such as c-reactive proteins [CRP], interlukins and tumor necrosis factor [TNF] alpha)^51^, which have been linked to the pathogenesis of T2DM. Third, the accumulation of visceral fat has been suggested to have lipolytic potential. This may, in turn, result in an increased delivery of free fatty acid to the liver’s portal circulation, which may induce hepatic insulin resistance by stimulating gluconeogenesis and interfering with hepatic insulin removal^50^.

Given that the relationship between total BFM and T2DM remain inconsistent, future studies are needed. While there is some evidence in studies using CT or MRI to measure subcutaneous fat^52^, data on the relationship between DXA-assessed SFM and T2DM are lacking. This is likely because previous versions of DXA were unable to compartmentalise SFM. Further studies using newer DXA models which can provide SFM in a large, population-based sample across the spectrum of BMI are warranted to assess the impact of SFM on the prevalence and incidence of T2DM. Studies unequivocally showed that people with T2DM were more likely to have lower MM^36–43^ through several mechanisms, such as decreased glucose utilization by muscle^53^, increased levels of systemic inflammatory cytokines such as interlukin-6, TNF-alpha, and C-reactive protein^54,55^ oxidative stress^56^, and mitochondrial dysfunction^57^. However, the relationship between reduced MM and risk of developing T2DM is still unclear^21,45,46^, and further studies are needed to determine this relationship and associated underpinning mechanisms.

There is substantial evidence indicating percent body fat differs between Asian and Western populations^58,59^, suggesting that the body composition profiles of patient with T2DM might also differ in these populations. As such, we also analysed various BC parameters separately in Asians and Caucasians. For BFM, most of the included studies (6 out of 8) were from Caucasians, limiting our ability to conclude whether objective fat measures differed between Asians and Caucasians. Of the 9 studies (4 Asians, 5 Caucasians) included in the systematic review of the DM-MM relationship, we found that T2DM was associated with low MM measures in both populations. However, there were only 3 studies (1 Asian, 2 Caucasians) for MM-DM relationship, with equivocal findings, suggesting the need for more studies to untangle this relationship in general, and between different populations.

There are several strengths of our systematic review. First, we only evaluated the relationship between BC measures and T2DM in human subjects, which allows for a more direct translation of results into clinical recommendations for patients with T2DM. Second, the studies in our review had wide geographic diversity, which can aid its generalizability. Third, we did not limit the timeframe, allowing a broad range of literature from 1998 to 2018. Finally, we excluded studies involving people with T1DM. This is important as there are pathophysiological, etiological, epidemiological and disease management differences between diabetes types which means that the influence of BC may differ between T1DM and T2DM.

While the majority of studies included in our review had high NOS scores, several limitations must be highlighted. First, eight studies were cross-sectional, which limit their ability to establish a causal relationship between BC and T2DM. The relatively low number of longitudinal studies (n = 5) included in the analysis, out of the 20 available in the literature, demonstrates the need for more well-designed cohort studies. Second, we excluded non-English publications which may have resulted in some publication bias. Third, as data from only eight articles were included in the meta-analysis, the generalizability of these findings might be limited. Fourth, the studies in our systematic review varied in their adjustment for potential confounders, thus we chose not to pool the adjusted odds ratio. Our meta-analysis summarised crude odds ratios, therefore our observed associations may be confounded by characteristics strongly related to T2DM risk that vary depending on body composition, such as diabetes duration and physical activity. Last, most studies only assessed a single BC parameter, and did not consider the interrelation between the key BFM measures (including both VFM and SFM) and MM. Future studies should assess the different BC phenotypes like low fat-high muscle mass, high fat-high muscle mass, low fat-low muscle mass, and high fat-low muscle mass to better reflect real world BC outcomes, which can then be translated into clearer clinical BC guidelines^60–64^.

In conclusion, our systematic review and meta-analysis demonstrated that higher BFM, particularly VFM, is associated with greater T2DM risk, and conversely the presence of T2DM was associated with a likelihood of lower MM. However, the relationship between MM (exposure) and T2DM remains unclear. Our findings suggest that optimal diabetes management and reducing VFM and increasing MM through lifestyle changes in the form of a more balanced diet and increased physical activity, may reduce the risk of T2DM and mitigate its deleterious impact on BC, respectively. However, further prospective studies to untangle the relationship between total BFM, SFM, and MM, on T2DM are needed in order to better inform clinical guidelines for disease prevention and management.
